# 2*H*-Chromen-4(3*H*)-one

**DOI:** 10.1107/S1600536811013535

**Published:** 2011-04-16

**Authors:** Richard Betz, Cedric McCleland, Harold Marchand

**Affiliations:** a Nelson Mandela Metropolitan University, Summerstrand Campus, Department of Chemistry, University Way, Summerstrand, PO Box 77000, Port Elizabeth 6031, South Africa

## Abstract

In the title compound, C_9_H_8_O_2_, a benzo-annulated heterocyclic ketone, the non-aromatic six-membered ring adopts an *E*_2_ conformation. In the crystal, C—H⋯O contacts connect the mol­ecules into double sheets perpendicular to the crystallographic *a* axis. The centroid–centroid distance for two π-systems is 3.7699 (6) Å.

## Related literature

For the structure of a chromium(0) compound containing the title compound as a ligand, see: Stewart *et al.* (1984 ▶). For graph-set analysis of hydrogen bonds, see: Etter *et al.* (1990 ▶); Bernstein *et al.* (1995 ▶). For puckering analysis, see: Cremer & Pople (1975 ▶).
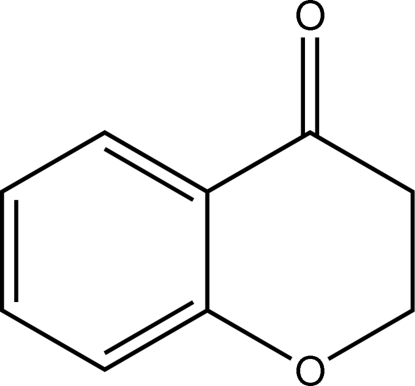


## Experimental

### 

#### Crystal data

C_9_H_8_O_2_
*M* *_r_* = 148.15
Monoclinic, 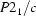

*a* = 7.5538 (4) Å
*b* = 8.0896 (4) Å
*c* = 13.0410 (6) Å
β = 115.364 (3)°
*V* = 720.08 (6) Å^3^
*Z* = 4
Mo *K*α radiation
μ = 0.10 mm^−1^
*T* = 200 K
0.48 × 0.41 × 0.29 mm

#### Data collection

Bruker APEXII CCD diffractometer
6367 measured reflections
1774 independent reflections
1578 reflections with *I* > 2σ(*I*)
*R*_int_ = 0.041

#### Refinement

*R*[*F* ^2^ > 2σ(*F* ^2^)] = 0.037
*wR*(*F* ^2^) = 0.107
*S* = 1.05
1774 reflections
100 parameters
H-atom parameters constrained
Δρ_max_ = 0.29 e Å^−3^
Δρ_min_ = −0.19 e Å^−3^

### 

Data collection: *APEX2* (Bruker, 2010 ▶); cell refinement: *SAINT* (Bruker, 2010 ▶); data reduction: *SAINT*; program(s) used to solve structure: *SHELXS97* (Sheldrick, 2008 ▶); program(s) used to refine structure: *SHELXL97* (Sheldrick, 2008 ▶); molecular graphics: *ORTEP-3* (Farrugia, 1997 ▶) and *Mercury* (Macrae,*et al.*, 2006 ▶); software used to prepare material for publication: *SHELXL97* and *PLATON* (Spek, 2009 ▶).

## Supplementary Material

Crystal structure: contains datablocks I, global. DOI: 10.1107/S1600536811013535/lw2060sup1.cif

Structure factors: contains datablocks I. DOI: 10.1107/S1600536811013535/lw2060Isup2.hkl

Additional supplementary materials:  crystallographic information; 3D view; checkCIF report

## Figures and Tables

**Table 1:** Hydrogen-bond geometry (Å, °)

*D*—H⋯*A*	*D*—H	H⋯*A*	*D*⋯*A*	*D*—H⋯*A*
C1—H11⋯O2^i^	0.99	2.53	3.4674 (13)	157
C1—H12⋯O2^ii^	0.99	2.56	3.3934 (15)	141

Symmetry codes: (i) 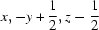; (ii) 
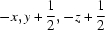
.

**Fig. 1.**
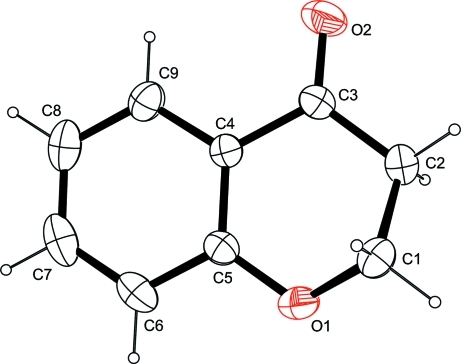
The molecular structure of the title compound, with atom labels and anisotropic displacement ellipsoids (drawn at 50% probability level).

**Fig. 2.**
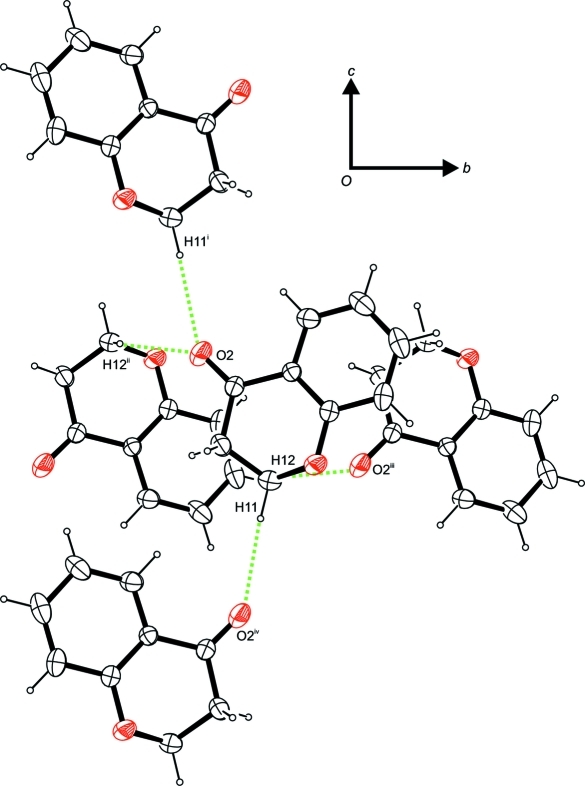
Intermolecular contacts, viewed along [-1 0 0]. Symmetry operators: ix, -y + 1/2, z + 1/2; ii -x, y - 1/2, -z + 1/2; iii -x, y + 1/2, -z + 1/2; ivx, -y + 1/2, z - 1/2.

**Fig. 3.**
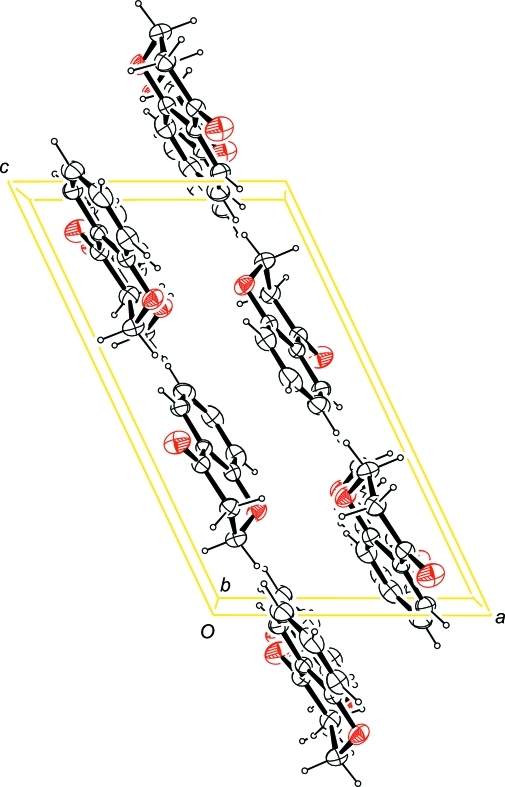
Molecular packing of the title compound, viewed along [0 1 0] (anisotropic displacement ellipsoids drawn at 50% probability level).

## supplementary crystallographic information

### Comment

In general, saturated, six-membered carbocycles and heterocycles adopt energetically favourable chair- or boat-conformations in solution and in the solid state although a variety of other conformations such as half-chair- or twist-forms are available. The annulation of aromatic rings can influence the conformation of such ring-systems and "freeze" one of the less common conformations. In our continued interest in effects of substituents and annulation of differently-substituted aromatic systems on the conformation of six-, seven- and eight-membered ring systems, we determined the crystal structure of the title compound to enable comparative studies. As of today, only one structural analysis of a chromium(0)-compound featuring the title compound as a ligand is apparent in the literature (Stewart *et al.* 1984).

The heterocyclic six-membered ring adopts an *E*_2_ conformation with carbon atom C1 acting as the *envelope*-atom. The latter one is displaced by 0.337 (1) Å from the least-squares plane defined by the atoms of the heterocycle. The least-squares planes defined by the carbon atoms of the phenyl ring as well as the skeletal atoms of the heterocycle intersect at an angle of 7.73 (6)°.

In the crystal structure, C–H···O contacts whose ranges fall by about 0.2 Å below the sum of van-der-Waals radii of the atoms participating are observed. These include both H atoms of the methylene group in *ortho*-position to the intracyclic O atom as donor atoms and exclusively the O atom of the carbonyl group as acceptor (Fig. 2). In total, the molecules are connected to double layers perpendicular to the crystallographic *a* axis. In terms of graph-set analysis (Etter *et al.*, 1990; Bernstein *et al.*, 1995) the descriptor for these contacts on the unitary level is *C*(5)*C*(5). The shortest *C*_g_···*C*_g_-distance for two π-systems was measured at 3.7699 (6) Å.

The packing of the title compound is shown in Figure 3.

### Experimental

The compound was obtained commercially (Aldrich). Crystals suitable for the X-ray diffraction study were taken directly from the provided product.

### Refinement

Carbon-bound H-atoms were placed in calculated positions (C—H 0.95 Å for aromatic C atoms and C—H 0.99 Å for aliphatic C atoms) and were included in the refinement in the riding model approximation, with *U*(H) set to 1.2*U*_eq_(C).

### Crystal data

**Table:**

C_9_H_8_O_2_	*F*(000) = 312
*M**_r_* = 148.15	*D*_x_ = 1.367 Mg m^−^^3^
Monoclinic, *P*2_1_/*c*	Melting point = 308–311 K
Hall symbol: -P 2ybc	Mo *K*α radiation, λ = 0.71073 Å
*a* = 7.5538 (4) Å	Cell parameters from 4401 reflections
*b* = 8.0896 (4) Å	θ = 3.0–28.3°
*c* = 13.0410 (6) Å	µ = 0.10 mm^−^^1^
β = 115.364 (3)°	*T* = 200 K
*V* = 720.08 (6) Å^3^	Block, colourless
*Z* = 4	0.48 × 0.41 × 0.29 mm

### Data collection

**Table:**

Bruker APEXII CCD diffractometer	1578 reflections with *I* > 2σ(*I*)
Radiation source: fine-focus sealed tube	*R*_int_ = 0.041
graphite	θ_max_ = 28.3°, θ_min_ = 3.5°
φ and ω scans	*h* = −9→10
6367 measured reflections	*k* = −10→10
1774 independent reflections	*l* = −17→14

### Refinement

**Table:**

Refinement on *F*^2^	Primary atom site location: structure-invariant direct methods
Least-squares matrix: full	Secondary atom site location: difference Fourier map
*R*[*F*^2^ > 2σ(*F*^2^)] = 0.037	Hydrogen site location: inferred from neighbouring sites
*wR*(*F*^2^) = 0.107	H-atom parameters constrained
*S* = 1.05	*w* = 1/[σ^2^(*F*_o_^2^) + (0.0588*P*)^2^ + 0.1339*P*] where *P* = (*F*_o_^2^ + 2*F*_c_^2^)/3
1774 reflections	(Δ/σ)_max_ < 0.001
100 parameters	Δρ_max_ = 0.29 e Å^−^^3^
0 restraints	Δρ_min_ = −0.19 e Å^−^^3^

### Fractional atomic coordinates and isotropic or equivalent isotropic displacement parameters (Å^2^)

**Table:**

	*x*	*y*	*z*	*U*_iso_*/*U*_eq_	
O1	0.32152 (11)	0.53935 (9)	0.22765 (6)	0.0337 (2)	
O2	0.15764 (13)	0.19047 (10)	0.39707 (7)	0.0434 (2)	
C1	0.20014 (15)	0.40212 (13)	0.16627 (8)	0.0327 (2)	
H11	0.2296	0.3735	0.1014	0.039*	
H12	0.0606	0.4348	0.1357	0.039*	
C2	0.23447 (15)	0.25263 (12)	0.24191 (8)	0.0307 (2)	
H21	0.1432	0.1632	0.1991	0.037*	
H22	0.3699	0.2120	0.2654	0.037*	
C3	0.20392 (13)	0.29406 (11)	0.34541 (8)	0.0262 (2)	
C4	0.23848 (12)	0.46883 (11)	0.38176 (7)	0.0232 (2)	
C5	0.29805 (12)	0.58227 (11)	0.32203 (8)	0.0255 (2)	
C6	0.33856 (14)	0.74544 (12)	0.35963 (9)	0.0349 (2)	
H6	0.3802	0.8228	0.3197	0.042*	
C7	0.31759 (15)	0.79357 (13)	0.45538 (10)	0.0400 (3)	
H7	0.3450	0.9047	0.4807	0.048*	
C8	0.25740 (16)	0.68291 (14)	0.51532 (9)	0.0389 (3)	
H8	0.2424	0.7180	0.5807	0.047*	
C9	0.21953 (14)	0.52099 (13)	0.47876 (8)	0.0310 (2)	
H9	0.1801	0.4441	0.5200	0.037*	

### Atomic displacement parameters (Å^2^)

**Table:**

	*U*^11^	*U*^22^	*U*^33^	*U*^12^	*U*^13^	*U*^23^
O1	0.0403 (4)	0.0338 (4)	0.0332 (4)	−0.0066 (3)	0.0217 (3)	0.0014 (3)
O2	0.0608 (5)	0.0309 (4)	0.0420 (4)	−0.0108 (3)	0.0254 (4)	0.0046 (3)
C1	0.0385 (5)	0.0353 (5)	0.0253 (4)	−0.0009 (4)	0.0146 (4)	−0.0024 (4)
C2	0.0348 (5)	0.0262 (4)	0.0318 (5)	−0.0008 (4)	0.0147 (4)	−0.0051 (4)
C3	0.0263 (4)	0.0239 (4)	0.0267 (4)	−0.0010 (3)	0.0098 (3)	0.0018 (3)
C4	0.0221 (4)	0.0233 (4)	0.0231 (4)	0.0013 (3)	0.0086 (3)	0.0007 (3)
C5	0.0234 (4)	0.0240 (4)	0.0279 (4)	−0.0001 (3)	0.0098 (3)	0.0012 (3)
C6	0.0318 (5)	0.0236 (5)	0.0446 (6)	−0.0032 (4)	0.0117 (4)	0.0013 (4)
C7	0.0329 (5)	0.0275 (5)	0.0474 (6)	0.0022 (4)	0.0057 (4)	−0.0114 (4)
C8	0.0374 (5)	0.0423 (6)	0.0314 (5)	0.0087 (4)	0.0095 (4)	−0.0107 (4)
C9	0.0313 (5)	0.0358 (5)	0.0256 (4)	0.0042 (4)	0.0121 (4)	0.0001 (4)

### Geometric parameters (Å, °)

**Table:**

O1—C5	1.3616 (11)	C4—C5	1.3970 (12)
O1—C1	1.4439 (12)	C4—C9	1.3977 (13)
O2—C3	1.2166 (12)	C5—C6	1.3957 (13)
C1—C2	1.5112 (14)	C6—C7	1.3803 (16)
C1—H11	0.9900	C6—H6	0.9500
C1—H12	0.9900	C7—C8	1.3870 (18)
C2—C3	1.5013 (13)	C7—H7	0.9500
C2—H21	0.9900	C8—C9	1.3813 (15)
C2—H22	0.9900	C8—H8	0.9500
C3—C4	1.4785 (12)	C9—H9	0.9500
			
C5—O1—C1	113.55 (7)	C5—C4—C3	120.27 (8)
O1—C1—C2	111.27 (8)	C9—C4—C3	120.43 (9)
O1—C1—H11	109.4	O1—C5—C6	117.70 (9)
C2—C1—H11	109.4	O1—C5—C4	122.30 (8)
O1—C1—H12	109.4	C6—C5—C4	119.99 (9)
C2—C1—H12	109.4	C7—C6—C5	119.45 (10)
H11—C1—H12	108.0	C7—C6—H6	120.3
C3—C2—C1	111.02 (8)	C5—C6—H6	120.3
C3—C2—H21	109.4	C6—C7—C8	121.35 (9)
C1—C2—H21	109.4	C6—C7—H7	119.3
C3—C2—H22	109.4	C8—C7—H7	119.3
C1—C2—H22	109.4	C9—C8—C7	119.13 (10)
H21—C2—H22	108.0	C9—C8—H8	120.4
O2—C3—C4	122.29 (9)	C7—C8—H8	120.4
O2—C3—C2	122.37 (9)	C8—C9—C4	120.82 (10)
C4—C3—C2	115.33 (8)	C8—C9—H9	119.6
C5—C4—C9	119.25 (9)	C4—C9—H9	119.6
			
C5—O1—C1—C2	−55.93 (11)	C3—C4—C5—O1	1.80 (13)
O1—C1—C2—C3	55.36 (11)	C9—C4—C5—C6	0.23 (13)
C1—C2—C3—O2	154.40 (10)	C3—C4—C5—C6	−177.25 (8)
C1—C2—C3—C4	−26.96 (11)	O1—C5—C6—C7	−179.64 (9)
O2—C3—C4—C5	177.76 (9)	C4—C5—C6—C7	−0.55 (14)
C2—C3—C4—C5	−0.88 (12)	C5—C6—C7—C8	0.14 (15)
O2—C3—C4—C9	0.32 (14)	C6—C7—C8—C9	0.60 (16)
C2—C3—C4—C9	−178.33 (8)	C7—C8—C9—C4	−0.93 (15)
C1—O1—C5—C6	−153.72 (9)	C5—C4—C9—C8	0.52 (14)
C1—O1—C5—C4	27.22 (12)	C3—C4—C9—C8	177.99 (8)
C9—C4—C5—O1	179.27 (8)		

### Hydrogen-bond geometry (Å, °)

**Table:**

*D*—H···*A*	*D*—H	H···*A*	*D*···*A*	*D*—H···*A*
C1—H11···O2^i^	0.99	2.53	3.4674 (13)	157
C1—H12···O2^ii^	0.99	2.56	3.3934 (15)	141

Symmetry codes: (i) *x*, −*y*+1/2, *z*−1/2; (ii) −*x*, *y*+1/2, −*z*+1/2.
